# β carbonic anhydrase is required for female fertility in *Drosophila melanogaster*

**DOI:** 10.1186/s12983-015-0111-3

**Published:** 2015-08-22

**Authors:** Leo Syrjänen, Susanna Valanne, Marianne Kuuslahti, Tea Tuomela, Ashwin Sriram, Alberto Sanz, Howard T. Jacobs, Mika Rämet, Seppo Parkkila

**Affiliations:** BioMediTech, University of Tampere, Tampere, Finland; School of Medicine, University of Tampere and Tampere University Hospital, Tampere, Finland; Department of Otorhinolaryngology, Central Finland Central Hospital, Jyväskylä, Finland; Institute for Cell and Molecular Biosciences and Newcastle University Institute for Ageing Health, Newcastle University, Newcastle upon Tyne, UK; Institute of Biotechnology, University of Helsinki, Helsinki, Finland; Tampere University Hospital, Tampere, Finland; Department of Pediatrics, Tampere University Hospital, Tampere, Finland; PEDEGO Research Center, Medical Research Center Oulu, and Department of Children and Adolescents, Oulu University Hospital, Oulu, Finland; Fimlab Laboratories Ltd, Tampere, Finland

## Abstract

**Background:**

Carbonic anhydrases (CAs, EC 4.2.1.1) are ubiquitous enzymes that catalyze the reversible hydration reaction of carbon dioxide. CAs are present as six structurally divergent enzyme families: α, β, γ, δ, ζ and η. β-CAs have a wide distribution across different species including invertebrates. Previously, we showed that *Drosophila melanogaster* β-CA is a highly active mitochondrial enzyme. In this study, we investigated the function of *Drosophila* β-CA by silencing the expression of the *β-CA* gene using UAS/GAL4-based RNA interference (RNAi) in *Drosophila* in vivo.

**Results:**

Crossing *β-CA* RNAi lines over ubiquitous *Actin* driver flies did not produce any viable progeny, indicating that *β-CA* expression is required for fly development. RNAi silencing of *β-CA* ubiquitously in adult flies did not affect their survival rate or function of mitochondrial electron transport chain. Importantly, *β-CA* RNAi led to impaired reproduction. All β-CA knockdown females were sterile, and produced few or no eggs. Whole ovaries of knockdown females looked normal but upon cadherin staining, there was an apparent functional defect in migration of border cells, which are considered essential for normal fertilization.

**Conclusions:**

These results indicate that although *Drosophila* β-CA is dispensable for survival of adult flies, it is essential for female fertility.

**Electronic supplementary material:**

The online version of this article (doi:10.1186/s12983-015-0111-3) contains supplementary material, which is available to authorized users.

## Background

Carbonic anhydrases (CAs, EC 4.2.1.1) are metalloenzymes that catalyze the reversible hydration reaction of carbon dioxide according to the following equation: CO_2_ + H_2_O ↔HCO_3_^−^ + H^+^ [1]. This reaction catalyzed by CAs is fundamental in the regulation of acid–base balance in living organisms. In addition, this reaction is involved in many physiological processes such as gluconeogenesis and ureagenesis, and it also helps to remove carbon dioxide out of tissues [1].

To date, six different classes of CAs have been identified: α, β, γ, δ, ζ and η [2, 3]. The three major classes α, β and γ are widely distributed among living organisms. On the other hand, the ζ-CAs are found only in diatoms, and the δ-CAs in diatoms and other marine phytoplankton. The novel group of CAs, namely η-CAs, was only recently discovered from malaria causing protozoan organisms of the genus *Plasmodium*. Of different CA-classes, β-CAs seems to be the class with the widest distribution. β-class CAs have been characterized throughout the tree of life. These enzymes are found in most species belonging to the *Archaea* and *Bacteria* domains and additionally probably all species of fungi and plants among domain *Eukarya* [4]*.* However*,* β-CAs are not present in in humans or other vertebrates [5].

β-CAs were first characterized in two metazoan organisms, namely the fruit fly *Drosophila melanogaster* [5]*,* which possesses one β-CA, CAHβ (with annotation symbol CG11967, flybase number FBgn0037646, also called DmBCA [5]) and the nematode *Caenorhabditis elegans* [6]*,* which possesses two β-CAs, one of which seems to be inactive. There have been no studies focusing on the biological function of β-CA in *D. melanogaster*. Fasseas and coworkers found no phenotypic changes in *C. elegans* when they performed RNAi experiments by feeding [6]. Recently, another β-CA was characterized from the protozoan parasite *Leishmania* and the effects of different CA-inhibitors were tested *in vitro* and also against living parasites in vivo. One of the studied compounds was a Schiff’s base type bromoderivative, which was a very efficient inhibitor of β-CA both *in vitro* and in vivo. Addition of this compound into growth medium containing living *Leishmania* parasites led to intracellular damages and death of the parasites [7]. Hence, it seems that β-CA is an essential enzyme for survival of *Leishmania* parasites.

The physiological roles of β-CAs are poorly understood, especially in invertebrate animal species having this gene. However, in some organisms β-CA has a support role for enzymes that utilize or dispose of CO_2_ or HCO_3_^−^. One example of such an enzyme is ribulose-1,5-biphosphate carboxylase (Rubisco) [8]. Even though *D. melanogaster* β-CA was characterized four years ago, the physiological significance of this enzyme has remained unclear. Here we have investigated the function of *Drosophila* β-CA utilizing RNA interference (RNAi) -mediated gene silencing in both developing and adult flies. We show that β-CA is needed for proper border cell migration in the developing egg and is therefore essential for egg fertilization.

## Results

### Ubiquitous silencing of *β-CA* causes lethality in *Drosophila* during development

**Fig. 1:**
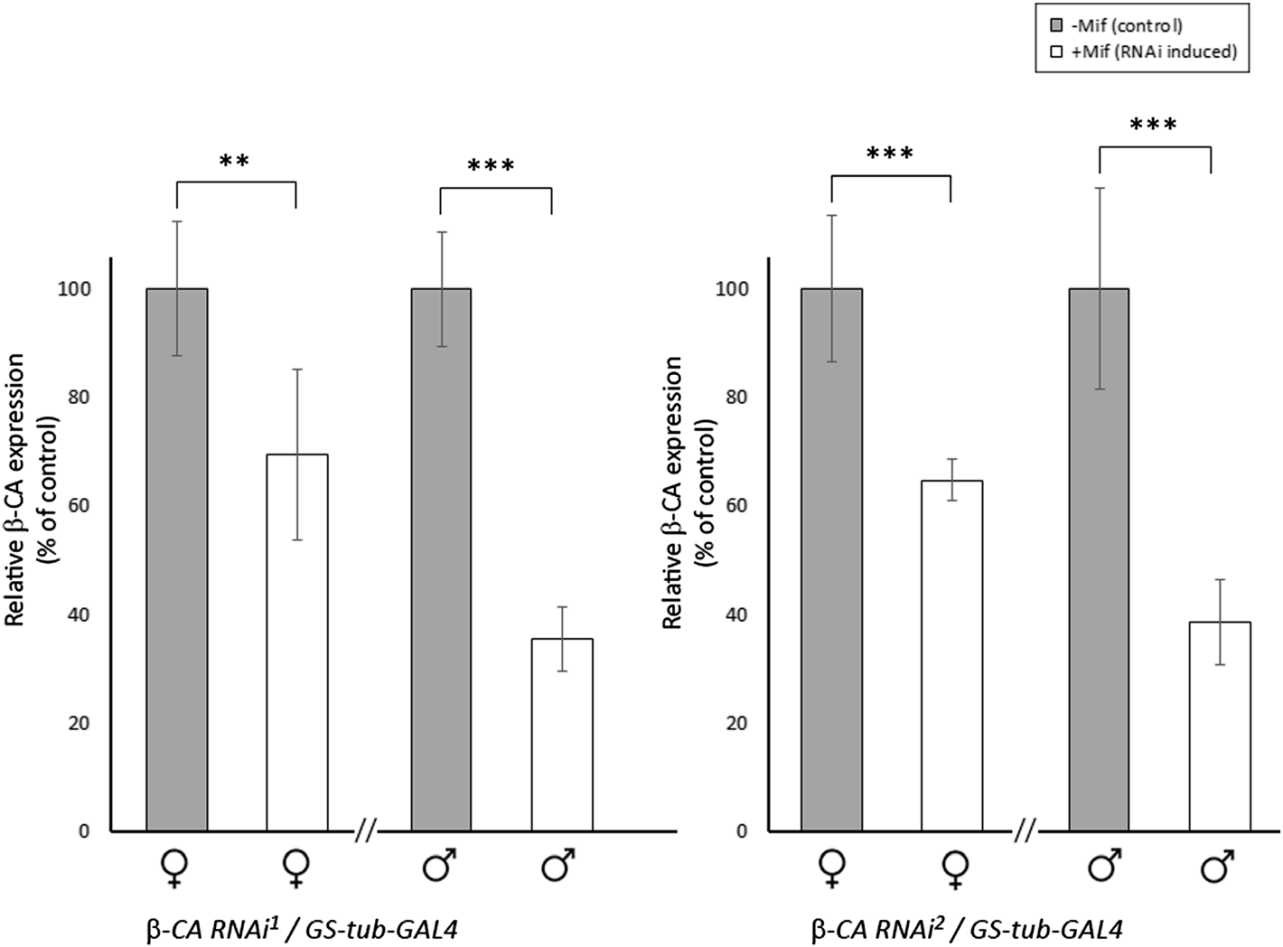
*β-CA* gene expression level is reduced in RNAi knockdown flies (+Mif) compared to control flies (−Mif). *β-CA* RNAi lines were crossed over the *GS-tub-GAL4* line and the eclosed F1 generation progeny was grown for 6 days at +29 °C in food with Mif (knockdown flies, +Mif) or without Mif (control flies, −Mif). Total RNA was extracted from 3 x 3–5 females and males of the lines indicated. Results are shown as % of expression of the control lines, which were normalized to 100 % each. ** = *p* < 0.01, *** = *p* < 0.001. *β-CA* RNAi^1^/*GS-tub-GAL4* females ctrl vs. RNA-induced: t stat = 3.63, df = 9; *β-CA* RNAi^1^/*GS-tub-GAL4* males ctrl vs. RNA-induced: t stat = 12.15, df = 9; *β-CA* RNAi^2^/*GS-tub-GAL4* females ctrl vs. RNA-induced: t stat = 6.13, df = 10; *β-CA* RNAi^1^/*GS-tub-GAL4* males ctrl vs. RNA-induced: t stat = 7.48, ds = 10.

Crossing two independent *β-CA* RNAi lines (#100233 and #38612, referred to as *β-CA* RNAi^1^ and *β-CA* RNAi^2^, respectively) over ubiquitous *ActGAL4*/CyOy + flies did not produce viable progeny of the phenotype indicative of *β-CA* dsRNA expression, implying that silencing of *β-CA* is lethal during fly development. In order to bypass developmental defects and to analyze the significance of *β-CA* expression for adult *Drosophila*, we used the inducible *GeneSwitch-tubulin-GAL4* (*GS-tub-GAL4*) driver line for *β-CA* silencing. The Gene Switch system allows selective expression of the hairpin construct in a chosen time of development, via addition of the inducing agent, Mifepristone (RU-486, hereafter Mif), in the fly food. Therefore, genetically identical siblings from a cross can be used as experimental flies (induction with Mif) and controls (no induction), which eliminates potential concerns regarding genetic background effects. Therefore, after eclosion, F1 progeny (*β-CA* RNAi/*GS-tub-GAL4*) flies were placed in Mif-containing food for *β-CA* silencing in the adults, and control siblings were placed in food without Mif. Firstly, the efficacy of gene silencing in *β-CA* RNAi flies crossed over *GS-tub-GAL4* and treated with Mif was analyzed using qRT-PCR. As shown in Fig. 1, both RNAi lines showed effective silencing when crossed over *GS-tub-GAL4* and induced with Mif. In *β-CA* RNAi^1^/*GS-tub-GAL4* males the level of gene expression was 35 ± 6 % from that of control males without Mif induction, and in females 69 ± 16 % when compared to control females without induction. With *β-CA* RNAi^2^/*GS-tub-GAL4* flies the values were 39 ± 8 % and 65 ± 4 %. These data indicate that Mif induced RNAi can be used to analyze the importance of *β-CA* expression in adult flies.

### *β-CA* expression is required for female fertility in *Drosophila*

**Fig. 2:**
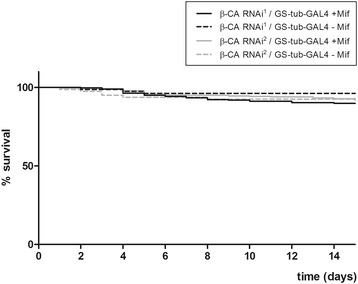
Fly survival is not impaired upon knockdown of *β-CA* expression. *β-CA RNAi* ^*1*^ and *β-CA RNAi* ^*2*^ lines were crossed over *GS-tub-GAL4. β-CA RNAi* ^*1*^/*GS-tub-GAL4* flies (*n* = 385) and *β-CA RNAi* ^*2*^/*GS-tub-GAL4* flies (*n* = 504) were collected and grown in food vials containing 400 μM Mif for 15 days. Flies from the same crosses were collected and used as controls by growing them in food vials without Mif (*n* = 81 and *n* = 80, respectively). The number of dead flies was recorded daily

To evaluate whether *β-CA* expression is required for survival of adult flies, *β-CA* RNAi flies were crossed to *GS-tub-GAL4* and monitored for survival after introduction to a Mif-containing diet. During 15 days follow-up, there were no statistically significant differences in survival between *β-CA* knockdown flies and controls: 90 % of *β-CA* RNAi^1^/*GS-tub-GAL4* F1 progeny flies were alive, while the percentage of live flies was 96 % in the control group. In the *β-CA* RNAi^2^/*GS-tub-GAL4* group the values were 92 % and 93 %, respectively (Fig. 2). This indicates that β-CA knockdown has no effect on the fly survival during the 15 day follow-up time, but does not exclude lifespan differences that may appear during later life. However, the analysis of fertility indicated that the egg laying capacity of Mif-induced *β-CA* RNAi/*GS-tub-GAL4* females was severely reduced. In one representative experiment performed, 50 female knockdown flies were kept together with 30 control *w*^*1118*^ males, and the flies did not lay a single egg within four days. Occasionally, a very low number of eggs were seen (Additional file 1: Table S1; 0–5 daily), but the eggs did not hatch. On the contrary, eggs, larvae and adult flies were found in all other cross combinations (Additional file 1: Table S1). In vials containing control *w*^*1118*^ females and *β-CA* RNAi/*GS-tub-GAL4* males, adult flies eclosed. This result implicated that *β-CA* has an essential function for the female fertility in *Drosophila*. Of note, fertility of female flies was restored by the second day after transferring the flies onto normal food. Thus, the effect on fertility was reversible.

### Mitochondrial oxygen consumption

**Fig. 3:**
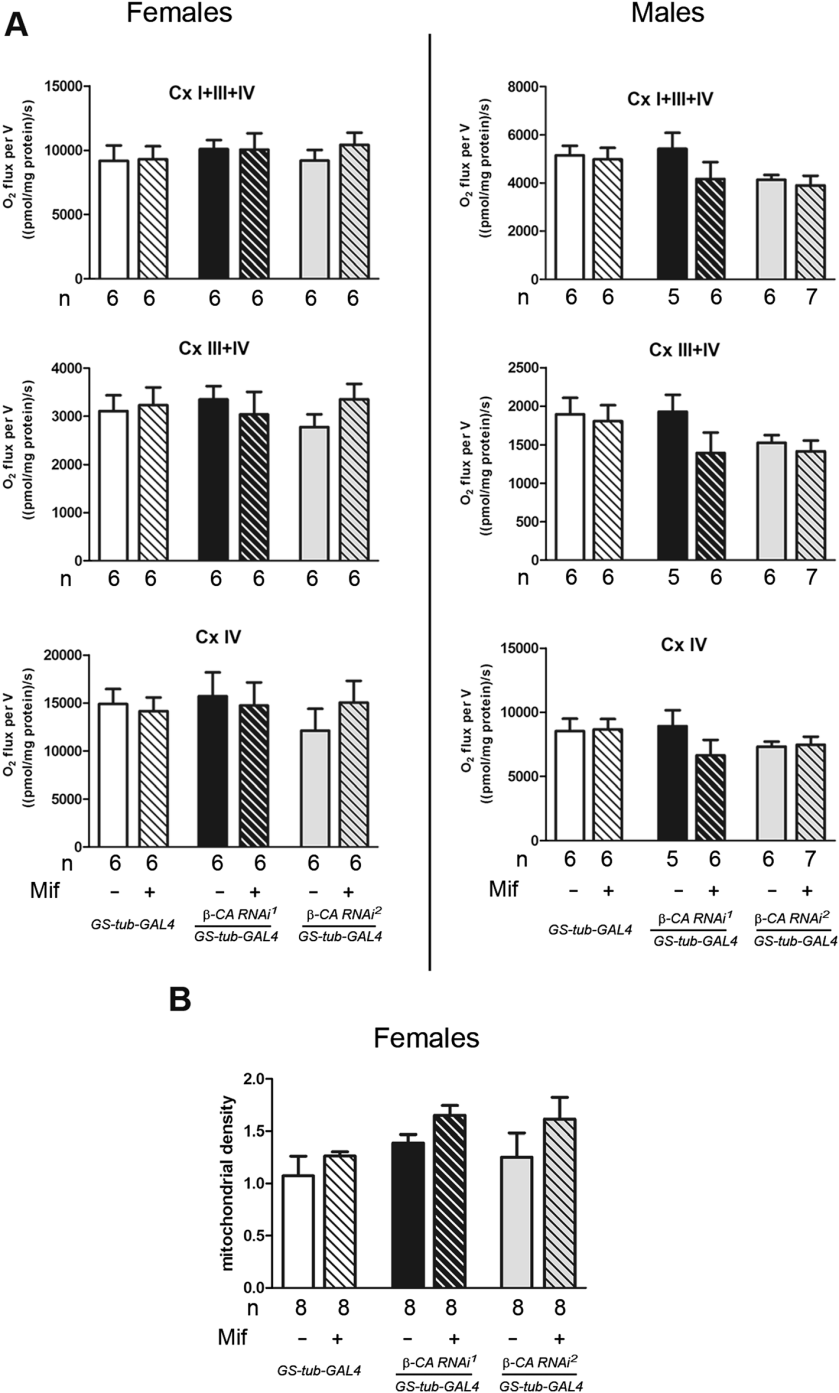
*β-CA* RNAi does not alter mitochondrial function in fruit flies. **a** Mitochondrial oxygen consumption (nmol O_2_/s mg protein) from female and male *Drosophila* flies using P + Pr + ADP (CI + III + IV), G3P (CIII + IV) and Ascorbate + TMPD (CIV) as substrates. Number of experiments (*n*) per group is indicated, data are shown as the mean ± SEM. **b**) Mitochondrial density measured as citrate synthase activity (UNITS mg/ml of mitochondrial protein) shown as the mean ± SEM from 8 replicate experiments. ADP - Adenosine diphosphate; G3P - Glyceraldehyde 3-phosphate; P – pyruvate; Pr – proline; TMPD - N,N,N',N'-Tetramethyl-*p-*Phenylenediamine dichloride

Since we have shown earlier that β-CA is a mitochondrial protein [5], we decided to assess whether silencing of *β-CA* gene expression affects mitochondrial oxygen consumption. Upon knockdown of *β-CA* we observed a female sterile phenotype (Additional file 1: Table S1); it has been recently shown that mutations in mitochondrial proteins cause general sterility in flies by dysfunction of the electron transport chain (ETC.) [9]. Moreover, complementation of the respiratory phenotype can rescue this and other associated phenotypes [9]. Using high resolution respirometry [10] we measured if mitochondrial respiration in whole fly preparations was affected upon knockdown of the *β-CA* gene. We did not observe any significant differences using complex I- (pyruvate + proline), complex III- (sn-glycerol-3-phosphate) or complex IV-linked (ascorbate + TMPD) substrates in the presence of ADP (Fig. 3a). Additionally, mitochondrial density, measured by the citrate synthase assay, was not altered by *β-CA* RNAi (Fig. 3b). In conclusion, ubiquitous silencing of the *β-CA* gene in adult flies did not affect the mitochondrial oxygen consumption in whole flies.

### *β-CA* RNAi causes delayed migration of border cells in *Drosophila* oogenesis

**Fig. 4:**
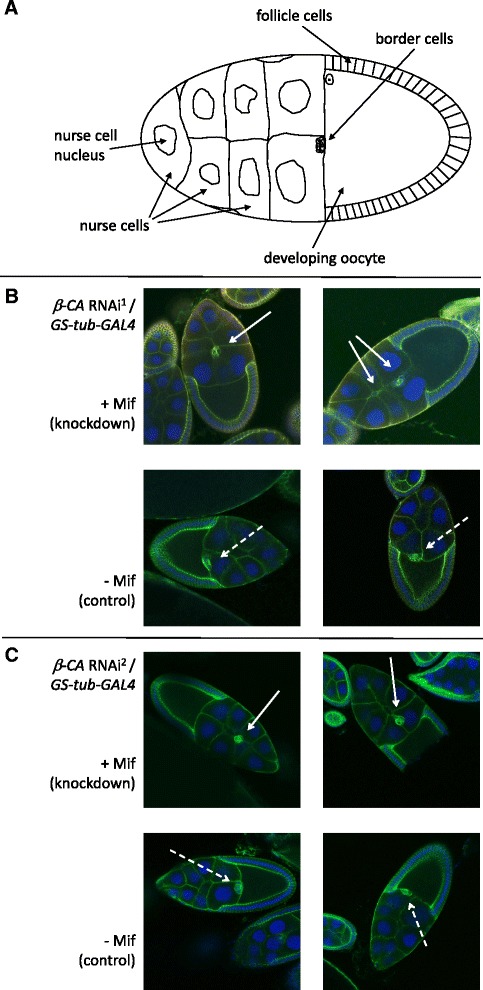
RNAi-mediated silencing of β-CA expression causes delayed border cell migration in *Drosophila* oogenesis. **a** A cartoon naming the cells involved in the development of an early stage 10 egg. **b** & **c**) Migration of DCAD2-labeled border cells towards the developing oocyte is delayed in *β-CA* knockdown early stage 10 eggs (white arrows) compared to controls (dashed white arrows). **b**) Developing eggs from *β-CA RNAi* ^*1*^/*GS-tub-GAL4* control and knockdown flies, **c**) Developing eggs from *β-CA RNAi* ^*2*^/*GS-tub-GAL4* control and knockdown flies

Because we discovered that silencing of *β-CA* causes female sterility by almost completely abolishing the egg laying capacity, we hypothesized that *β-CA* knockdown might cause a functional defect in the ovaries. To test this hypothesis, we exposed one to two-day old *β-CA* RNAi^1^/*GS-tub-GAL4* and *β-CA* RNAi^2^/*GS-tub-GAL4* female flies to Mif-containing diet to silence *β-CA* expression. Flies from the same crosses without Mif were used as controls. At day four, flies were exposed to males to facilitate egg production and after 6 days, ovaries from *β-CA* RNAi/*GS-tub-GAL4* knockdown and control flies were dissected. Ovaries were immunostained with DCAD2 antibody and observed under Carl Zeiss LSM 780 confocal microscope. DCAD2 antibody stains DE-cadherin in border cells, which are a group of somatic cells that migrate at stage 9 during *Drosophila* oogenesis. Border cells arise and detach from the monolayer follicular epithelium, and they invade and migrate between the nurse cells towards the oocyte. Border cells were so named because they end up on the border between nurse cells and oocyte at early stage 10 [11]. A cartoon of a normal, early stage 10 developing egg is shown in Fig. 4a. Representative images of early stage 10 developing oocytes in *β-CA* knockdown (+Mif) and control (−Mif) ovaries are shown in Fig. 4b (*β-CA* RNAi^1^ line) and 4c (*β-CA* RNAi^2^ line). The results clearly indicate that border cell migration was delayed in *β-CA* knockdown cells compared to controls. At early stage 10, border cells reached the border of the developing oocyte in controls, but in many of the knockdown ovaries the border cells were still under migration and located between the nurse cells. 14/36 *β-CA* RNAi^1^/*GS-tub-GAL4* early stage 10 knockdown oocytes showed delayed border cell migration, whereas in all 18 control oocytes analyzed, the border cells had already reached the border of the developing oocyte. The phenotype was weaker but visible also with the *β-CA* RNAi^2^/*GS-tub-GAL4* flies, where 7/27 knockdown oocytes showed delayed border cell migration compared to 26 controls, in all of which the movement of border cells was normal.

We conclude that silencing *β-CA* expression in *Drosophila* females causes delayed border cell migration during oogenesis, which contributes to the sterility of the female *β-CA* knockdown flies.

## Discussion

Our results show that β-CA is essential for *D. melanogaster* reproduction. RNAi-mediated silencing of the *β-CA* gene caused sterility of females. When *Drosophila* ovaries were examined from both control and RNAi flies, it was found that RNAi flies had disturbances in border cell migration during oogenesis. Previously it has been shown that defective border cell migration leads to sterility of female flies due to impaired formation of the micropyle which is needed in fertilization [12, 13]. Additionally, even mature virgin *Drosophila* females should spontaneously ovulate at low rate (~1 egg/day) [14], but the knockdown flies typically did not lay any eggs in our study (Additional file 1: Table S1). The mechanism by which oocytes are released from the *Drosophila* ovary is unknown. The absence of β-CA possibly affects the physiological conditions of the ovulatory tract so that ovulation does not occur. Expression of *β-CA* has been studied in a publicly available DNA microarray dataset: in FlyAtlas (http://flyatlas.org/atlas.cgi?name=CG11967-RA website, [15]) *β-CA* expression levels are provided for 17 adult and 8 larval *D. melanogaster* tissues. The highest upregulated *β-CA* expression levels in adult flies are found in spermatheca (female), fat body, and heart. The spermatheca is a sperm storage organ, and it is possible that, in addition to disturbances in border cell migration and to the possible effect on ovulation, female sterility upon *β-CA* knockdown is related to the function of spermatheca. Also, it is interesting that the reduced fertility of *β-CA* knockdown flies is reversible once *β-CA* gene expression is no longer silenced. This may be related to the fact that RNAi does not fully remove gene function but drastically lowers the *β-CA* expression dose, which is sufficient to cause the phenotype, but is reversible once normal gene expression is restored.

Although phenotypes caused by *β-CA* silencing (lethality during development and sterility in adult flies) are reminiscent of the ones caused by mitochondrial dysfunction [9], we did not detect alterations in electron transport chain (ETC.) functions. In the past, we have found that reduction in mitochondria respiration causes sterility, whereas complementation of the respiratory defect rescues it [9, 10, 16]. Here, the sterile phenotype was not associated with a reduction in respiration indicating a more subtle alteration in mitochondrial function.

Human isozymes CA IX and XII have been linked to tumor invasiveness and migration [17, 18]. It has been suggested that CAs facilitate cell migration by low pH-induced activation of matrix metalloproteinases [19]. Notably, these CA enzymes are membrane-bound, whereas β-CA is mitochondrial. Because HCO_3_^−^ does not readily diffuse across the mitochondrial membrane, the impaired movement of border cells probably results from disturbances in biosynthetic pathways. Interestingly, a mitochondrial β-CA has been shown to be important in the sexual reproduction of filamentous ascomycete *Sordaria macrospore* [20]. In the presence of *β-CA* gene mutation *cas2,* vegetative growth, fruiting-body development and ascospore germination were affected, and the double mutant strain *cas1/2* was completely sterile. In *S. macrospore*, *cas2* was shown to be mitochondrial while other two β-CAs *cas1* and *cas3* were cytoplasmic. Defects caused by the lack of *cas2* could be partially compensated by elevated carbon dioxide levels in addition to overexpression of *cas1*, *cas3*, or a non-mitochondrial *cas2* variant. It was depicted that there may be a functional connection between β-CAs and adenylyl cyclase. In this way, the cAMP signaling may be affected. However, this is related to carbon dioxide and bicarbonate sensing in fungi and therefore cannot be directly related to signaling pathways in animals.

The effect of mitochondrial CA function has been tested in mammals. In a recent study, the roles of CA VA and VB were examined in mice with targeted mutagenesis [21]. During the study it was found CA VA null mice were smaller than wild-type mice and bred poorly. However, when sodium-potassium citrate-supplemented water was given, the production of offspring was normal. Blood ammonia concentrations of CA VA null mice were elevated, but fasting blood sugar levels were normal. On the other hand, CA VB null mice showed normal growth, normal blood ammonia levels as well as normal fasting blood sugar levels. CA VA/B double-knockout (DKO) mice showed additional abnormalities. Impairment of growth and hyperammonemia were more severe than for CA VA null mice. DKO animals reproduced less than predicted despite of supplemented sodium-potassium citrate in their drinking water. Additionally, survival after weaning was reduced, especially for males. Moreover, fasting blood glucose levels for DKO mice were significantly lower compared to controls. Clearly, these enzyme deficiencies in mice (and probably other vertebrates) are not lethal but cause significant metabolic problems and also have effects on breeding.

Fasseas and coworkers performed CA RNAi studies with *C. elegans* [6]. Two *β-CA* genes were found: *bca-1* and *y116a8c.28*. Of these, the latter was shown to encode for an active enzyme. In normal conditions, the authors found some phenotypes, like slow growth rates, but they were unable to consistently reproduce the effects, and the conclusion was that visible phenotype was not found. The level of gene silencing was not reported; it is possible that the level of silencing was not sufficient. On the other hand, it is possible that some other CA might compensate the loss of β-CA function. In addition to the results of this study, our recent findings on *Leishmania* parasites indicate that the enzyme is essential for invertebrates and protozoans [7].

*D. melanogaster* β-CA is a mitochondrial enzyme as are most of the other *Dipteran* β-CAs [22]. It is plausible that findings in *D. melanogaster* can be generalized also to the other *Dipteran* species which act commonly as disease vectors. These species include the malaria mosquito *Anopheles* and the yellow fever mosquito *Aedes.* In light of this, inhibitors against these enzymes could also be used to restrict the spread of various diseases.

## Conclusions

Thus far the physiological role of invertebrate β-CAs has been unclear. Our study indicates that the β-CA function is not vitally important for adult *D. melanogaster* since *β-CA* RNAi had no effect on survival, although the enzyme was required during development. However, β-CA is essential for reproduction. Our study suggests that disturbance of β-CA function leads to abnormal border cell migration during *Drosophila* oogenesis. Previously it has been shown that defective border cell migration leads to sterility of female flies due to impaired formation of micropyle which is needed in fertilization. Vertebrates do not possess β-class CAs, but these enzymes are widespread throughout the phylogeny of life on Earth. This makes them exciting new targets for parasitic drug development. Indeed, β-CAs are found in many pathogenic organisms and pathogen vectors of the animal kingdom and protozoans, including the *Leishmania* parasites, the malaria mosquito *Anopheles*, and the yellow fever mosquito *Aedes*. Our study shows that invertebrate β-CAs are indeed important enzymes, which encourages further studies on anti-parasitic drug development. Since interference of β-CA function seems to cause sterility of female flies, this feature could be used in controlling the amount of insects that cause or distribute harmful diseases.

## Methods

### Generation of β-CA knockdown flies

Two different *β-CA* RNAi lines were obtained from Vienna *Drosophila* RNAi Center (VDRC) from KK and GD collections with the following IDs: #100233 (hereafter referred to as *β-CA RNAi*^*1*^*)* and #38612 (hereafter referred to as *β-CA RNAi*^*2*^*)* [23]. These stocks have been generated to overexpress a dsRNA hairpin construct, specific to the *β-CA* gene under *upstream activation sequence* (*UAS*) control. When these flies are crossed over a GAL4 activator protein expressing line, *β-CA* gene is silenced in the tissue where GAL4 protein is expressed [24]. For ubiquitous RNAi-mediated silencing, β-CA RNAi lines were crossed over the *Actin-GAL4* line or a Mifepristone-inducible *Geneswitch-tub5-GAL4* line [25].

### RNA extraction

*β-CA* RNAi lines were crossed over the *GS-tub-GAL4* line and the eclosed F1 generation progeny were grown for 6 days at +29 °C in food with 400 μM Mif (knockdown flies) or without Mif (control flies). The flies were transferred into new food vials once during the period. RNA extractions were made as triplicates for both sexes: for each line, 3 x 3–5 females and males were used. RNA extraction was done using TRI Reagent^®^ Solution (Ambion), according to manufacturer’s instructions.

### Quantitative reverse transcriptase PCR (qRT-PCR)

qRT-PCR was performed with the above mentioned extracted total RNAs to quantify the level of gene silencing. Also RNA extracted from the *GS-tub-GAL4* line was tested by qRT-PCR to identify possible leakiness of the tubulin driver. PCR reactions were performed in MicroAmp optical 96-well reaction plates using a SYBR-Green PCR master mix kit (Applied Biosystems), according to the manufacturer's instructions. Primers for qRT-PCR were designed using Primer Express Software v2.0 (Applied Biosystems). The forward primer used in the reaction for β-CA gene expression was 5’-GACAAGGGAGCAAATGGTCAA-3’ and the reverse primer was 5’-TCTACTGTCCATGCAGGTGAAGAA-3’. The β-CA amplicon size was 88 bp. The reaction was carried out in an ABI PRISM 7000 Detection System (Applied Biosystems). The data were analyzed with ABI PRISM 7000 SDS software and normalized to the RpL32 housekeeping gene. The forward primer used for RpL32 was 5’-GGTTACGGATCGAACAAGCG-3’ and the reverse primer was 5’-TTCTGCATGAGCAGGACCTC-3’. The RpL32 amplicon size was 101 bp. The final results were expressed as % of expression of the control lines, which were normalized to 100 % each, as described in [26].

### Survival study

To study the effect of the *β-CA* gene knockdown on the survival rate of flies, the *β-CA RNAi*^*1*^ and *β-CA RNAi*^*2*^ lines were crossed over *GS-tub-GAL4*. Eclosed, one day old *β-CA RNAi*^*1*^/*GS-tub-GAL4* flies and *β-CA RNAi*^*2*^/*GS-tub-GAL4* flies were collected and grown in mixed-sex groups in food vials containing 400 μM Mif for 15 days. Flies from the same crosses were collected and used as controls by growing them in food vials without Mif. The number of dead flies was recorded daily.

### Fly fertility

To study the fertility of *D. melanogaster* β-CA knockdown flies, the *β-CA RNAi*^*1*^/*GS-tub-GAL4* and *β-CA RNAi*^*2*^/*GS-tub-GAL4* eclosed flies were collected and placed at +29 °C in normal food (control) or food containing 400 μM Mif (*β-CA RNAi* induced) for six days. *w*^*1118*^ control flies were kept in the same conditions. Thereafter the flies were mated with flies from the same cross or with *w*^*1118*^ control flies, in different combinations (Additional file 1: Table S1). Fertility was followed up to 15 days.

Additionally, the possible reversibility of fly fertility was studied by providing normal food to the previously mentioned flies after 6 days. The flies were put into new vials daily to find out when their fertility was restored.

### Measurement of mitochondrial oxygen consumption by high-resolution respirometry

Whole fly homogenates from *β-CA RNAi*^*1*^/*GS-tub-GAL4* and *β-CA RNAi*^*2*^/*GS-tub-GAL4*, grown with or without Mif for six days, were used for respirometry measurements. Twenty flies were homogenized in MIB (250 mM sucrose, 2 mM EGTA, 5 mM Tris–HCl pH 7.4) and filtered before immediately being used in an OROBOROS O2k oxygraph (Oroboros Instruments, Innsbruck, Austria). Homogenates were incubated in assay buffer (120 mM KCl, 5 mM KH2PO4, 3 mM Hepes, 1 mM EGTA, 1 mM MgCl2, 0.2 % BSA, pH 7.2) at 25 °C. Experiments were performed according to the previously described protocol [10]. Values were normalized to protein concentration as calculated by the Bradford method.

### Mitochondrial density measurements via the citrate synthase assay

Approximately 40–60 flies were immobilised on ice and then transferred to a chilled mortar. The flies were homogenised in 500 μl of ice-cold mitochondria isolation medium (250 mM sucrose, 5 mM Tris–HCl, 2 mM EGTA), and the homogenate was filtered through cheesecloth. Then, an additional 500 μl of the mitochondria isolation medium containing 1 mM DTT was added, and the samples were frozen at −80 °C overnight. Next, the samples were defrosted, and 50 μl of the sample was diluted 1:5 in mitochondria isolation medium containing 1 mM PMSF. The remainder of the sample was used to isolate mitochondria as described elsewhere [27]. The mitochondria were subsequently diluted 1:4 in mitochondria isolation buffer containing 1 mM PMSF. Measurements were performed in a 96-well plate, in which 182 μl of fresh reaction buffer (100 mM Tris–HCl (pH 7.5) and 2.5 mM EDTA), 2 μl of 30 mM acetyl-CoA and 2 μl of 10 mM DTNB were added to each well. Finally, the samples (either the whole homogenate or isolated mitochondria) were added. The reaction was initiated by adding 10 μl of 10 mM oxaloacetate (OAA), and the linear increase in absorbance at 412 nm was followed for 3–4 min using a PerkinElmer EnVision 2104 plate reader. Blanks were made from the same samples without the addition of OAA and then measured. Mitochondrial density was calculated by dividing the specific citrate synthase activity measured in the whole-fly homogenates by the specific citrate synthase activity measured in isolated mitochondria.

### Dissection, staining and examination of ovaries

One to two-day old female *β-CA RNAi*^*1*^/*GS-tub-GAL4* and *β-CA RNAi*^*2*^/*GS-tub-GAL4* flies were placed at +29 °C in normal food (control) or food containing 400 μM Mif (*β-CA RNAi* induced) for six days. Flies were moved to fresh food with or without Mif every two days. On day four, male *Oregon R* flies were added to induce egg production of the females. On the 6^th^ day, the knockdown and control females were anesthetized on a CO_2_ pad and the ovaries were dissected.

The dissected ovaries were transferred in 0.5 ml tubes containing phosphate-buffered saline (PBS), fixed using 4 % paraformaldehyde (PFA) for 20 min at +4 °C and rinsed 2x with PBS 0.3 % Triton (PBT). Ovaries were washed 2x 15 min in PBT containing 0.5 % bovine serum albumin (BSA), incubated overnight with the DCAD2 primary antibody (Developmental Studies Hybridoma Bank, Iowa, USA, 1:20) in PBT-BSA, rinsed 2x with PBT and washed again 2 x 15 min in PBT-BSA. Incubation with the secondary Goat anti-rat antibody (AlexaFluor 488 conjugate, Life Technologies, 1:1000) in PBT-BSA was carried out for 2 h at RT. Ovaries were rinsed again 2x in PBT and washed 3x 15 min in PBT. DAPI (final conc. 1 μg/ml, Sigma) was added to the second last wash. After the final wash, ovaries were placed in 70 % glycerol for at least 30 min at +4 °C, and then transferred in Vectashield Mounting medium (Vector Laboratories).

Prior to microscopy, the ovaries were put on microscope slides, the egg chambers separated using a sharpened wire and the samples mounted and sealed using cover slips and nail varnish. Ovaries were examined and imaged using the Zeiss LSM 780 confocal microscope with 20x objective. Early stage 10 developing eggs were distinguished from earlier developmental stages based on the shape of the follicle cells surrounding the developing oocyte (see Fig. 4a and [28] Fig. 1b). Images are snap-shots of the focal plane of each developing oocyte where border cells are visible.

### Statistical analyses

Statistical analysis of *β-CA* gene expression in control and knockdown flies by qRT-PCR was carried out using Student’s *t*-test for two samples assuming equal variances. Statistical analysis of fly survival experiments was carried out in Prism 6 (GraphPad) using the log-rank (Mantel-Cox) test. High resolution respirometry data were analyzed with Prism 6 (GraphPad) using the unpaired Student’s *T*-test. The level of statistical significance was established as *p* < 0.05.

## Additional file

Additional file 1: Table S1. Fly fertility is severely reduced in crosses where *β-CA* is knocked down in females.
