# Supplementary material for: β carbonic anhydrase is required for female fertility in Drosophila melanogaster
Source: Front Zool. 2015 Aug 22;12:19. doi: 10.1186/s12983-015-0111-3 (PMC4546311; doi:10.1186/s12983-015-0111-3)
Supplement: Additional file 1: Table S1. — Fly fertility is severely reduced in crosses where β-CA is knocked down in females. [file 12983_2015_111_MOESM1_ESM.docx]

**Table 1. Fly fertility is severely reduced in crosses where *β-CA* is knocked down in females.** Both *β-CA RNAi^1^*/*GS-tub-GAL4* and *β-CA RNAi^2^*/*GS-tub-GAL4* flies were used in this experiment, with identical results.

+ = tens or hundreds found daily, (+) = 0-5 found daily, - = none found.

| **Cross** | | **Eggs** | | **Larvae** | | **Pupae** | | **Adult flies** | |
| --- | --- | --- | --- | --- | --- | --- | --- | --- | --- |
| **female** | **male** | **normal food** | **400 µM Mif** | **normal food** | **400 µM Mif** | **normal food** | **400 µM Mif** | **normal food** | **400 µM Mif** |
| ***w^1118^*** | ***w^1118^*** | + | + | + | + | + | + | + | + |
| *β-CA* ***RNAi / GS-tub-GAL4*** | ***w^1118^*** | + | (+) | + | - | + | - | + | - |
| ***w^1118^*** | *β-CA* ***RNAi / GS-tub-GAL4*** | + | + | + | + | + | + | + | + |
| *β-CA* ***RNAi / GS-tub-GAL4*** | *β-CA* ***RNAi / GS-tub-GAL4*** | + | (+) | + | - | + | - | + | - |
